# Comparative analysis of survival between older and nonolder severe sepsis and septic shock resuscitated patients

**DOI:** 10.1186/cc12661

**Published:** 2013-06-19

**Authors:** H Palomba, TD Correa, MSC Assunção, A Pardini, DR de Melo, E Silva

**Affiliations:** 1 Hospital Israelita Albert Einstein, Morumbi, São Paulo, SP, Brazil

## Introduction

Advanced age has been associated with increased mortality in severe sepsis and septic shock patients [1,2]. However, the impact of early resuscitation following the Surviving Sepsis Campaign Guidelines in this population of patients is unclear. The objective of this study was to compare the in-hospital mortality between older (EP) and nonolder (N-EP) resuscitated patients according to the Surviving Sepsis Campaign Guidelines.

## Methods

A retrospective observational study. All patients with severe sepsis and septic shock admitted to the ICU between January 2006 and March 2012 were studied. Comparisons were performed between older (≥65 years) and nonolder patients (<65 years).

## Results

**Figure 1:**
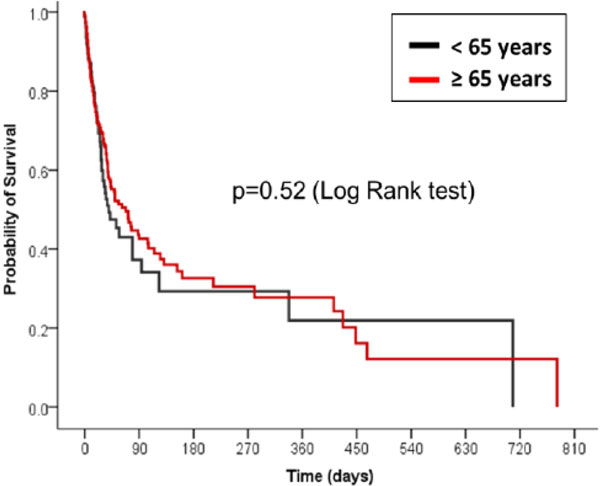
**Survival analysis comparing older and nonolder patients**.

A total of 913 patients with severe sepsis and septic shock were included in this analysis. Older patients accounted for 63% (573/913) of patients and nonolder patients for 37% (340/913) of patients. The median (IQR) age was 80 years (73 to 85) and 51 years (40 to 59) for EP and N-EP, respectively. The incidence of severe sepsis (43% vs. 44%) and septic shock (57% vs. 56%) did not differ between the EP and N-EP groups (*P *= 0.78). EP patients had a higher median (IQR) APACHE II score (23 (18 to 28)) than N-EP patients (19 (16 to 24), *P <*0.001), although the median number of organ dysfunctions (3 vs. 2 for EP and N-EP, respectively, *P *= 0.57) did not differ between the groups. EP patients were more likely to have hypertension (51% vs. 29%, *P *<0.001), diabetes (33% vs. 24%, *P *= 0.02), ischemic heart disease (16% vs. 7%, *P *<0.001) and chronic renal failure (8.5% vs. 4.2%, *P *<0.03) when compared with N-EP patients. Solid organ transplantation (24% vs. 4%, *P *<0.001) and liver cirrhosis (17% vs. 5%, *P *<0.001) were more frequently in N-EP patients. There was no significant between-group difference in the in-hospital mortality (33% in the EP group and 28% in the N-EP group; odds ratio, 1.27; 95% CI, 0.94 to 1.70; *P *= 0.12) (Figure 1). The length of hospital stay (14 (7 to 29) vs. 12 (6 to 21) days (median (IQR)), *P *= 0.001) was significantly higher in EP patients compared with the N-EP patients.

## Conclusion

In this population of severe sepsis and septic shock patients, the early resuscitation of older patients was not associated with increased mortality. However, prospective studies addressing the long-term impact of the resuscitation maneuvers on outcomes are necessary.
